# Effect of continuous passive motion on the early recovery outcomes after total knee arthroplasty

**DOI:** 10.1007/s00264-021-05245-5

**Published:** 2021-10-21

**Authors:** Magdalena Richter, Tomasz Trzeciak, Małgorzata Kaczmarek

**Affiliations:** Department of Orthopedics and Traumatology, Poznan University of Medical Sciences, 28 Czerwca 1956 r. 135/147, 61-545 Poznan, Poland

**Keywords:** Continuous passive motion, Total knee arthroplasty, Osteoarthritis, Rehabilitation

## Abstract

**Introduction:**

Continuous passive motion (CPM) is a frequently used method in the early post-operative rehabilitation of patients after knee surgery. In this study, the effectiveness of the CPM method was evaluated after primary total knee arthroplasty during an early recovery period.

**Methods:**

Eighty patients undergoing total knee arthroplasty were assigned into two groups. The experimental group received CPM and active exercises, while the control group active exercises only. All subjects were evaluated once before the surgery and at a discharge, in terms of mean active range of motion (AROM), mean Knee Society Score (KSS), and Western Ontario and MacMaster Universities Osteoarthritis Index (WOMAC).

**Results:**

The mean AROM for the experimental group was 82.3° ± 14.3° and 76.1° ± 22.2° for the control. The mean KSS score was 136.4 ± 19.3 points for the experimental group, and 135.7 ± 15.1 for the control. There were no statistical differences between the two groups. The KSS functional score was 66.4 ± 8.1 points for the experimental group compared to 62.2 ± 7.3 points for the control, but there was a statistically significant difference between the groups at discharge from the hospital (*p* = 0.009). A subjective estimation of the pain level, joint stiffness and function also showed a statistically significant difference between the two groups (38.6 ± 14.3 points for the CPM group and 21.2 ± 15.7 for the control).

**Conclusion:**

These findings show that there is no significant effect of CPM in terms of improving clinical measurements. However, there was a significant beneficial effect on the subjective assessment of pain level, joint stiffness, and functional ability.

## Introduction

Total knee arthroplasty (TKA) proved to be an excellent option for patients suffering from end-stage osteoarthritis [1]. However, outcomes following TKA depend on many aspects, such as the adequacy of physiotherapy and subsequent functional recovery. There are several methods to accelerate the patients’ recovery in an early post-operative (post-op) period. One of these methods is continuous passive motion (CPM) [2]. Salter [3], who first introduced CPM in the 1970’s, using the rabbit experimental model, demonstrated the beneficial role of early movement for recovery after a joint injury. Since then, CPM has been widely used in patients as an adjunct to conventional physiotherapy after TKA. However, controversies still exist as to whether it is useful/beneficial for patients’ recovery. For the past two decades, numerous studies have been carried out on the post-op efficiency of CPM [4–7]. Some studies recommend CPM, whereas others found contradictory results. The reported benefits included reduced swelling, faster recovery of flexion, decreased analgetics use, and shorter hospital stay [8–11]. However, differences in the study design, including the duration of CPM application per day, flexion regime, and duration of hospital stay, impede a definite conclusion concerning the advantages of CPM use in the early post-op period.

The objective of this study was to determine the effect of CPM on very early post-op outcomes in patients after TKA compared to outcomes after standard physiotherapeutic treatment. The outcome measurements included active range of motion (AROM), clinical and functional evaluation according to the Knee Society Score (KSS), and subjective functional measure using Western Ontario and McMaster Universities Osteoarthritis Index (WOMAC).

## Material and methods

Ninety-three patients were initially recruited in the study. Thirteen patients met the exclusion criteria (rheumatoid arthritis, osteotomy, post-traumatic osteoarthritis) and were subsequently excluded from the study. Results were analyzed for the remaining eighty patients, all diagnosed with primary OA. They were admitted to the Department of Orthopedics and Traumatology and underwent total knee arthroplasty. The procedure was performed by one of four experienced orthopaedic surgeons. After the TKA, patients from the study group underwent standard physiotherapeutic treatment with the addition of CPM, in the early post-op period (days 1 to 10). The CPM was implemented daily for two hours, starting with 30°–45° flexion, and the angle was increased by 10°–15° a day, based on the patients tolerance.

The control group received standard physiotherapy program, which started on post-op day one. In both groups, the program included respiratory and isometric exercises, active-assisted ROM exercises, transfer training, and walking.

Each patient from both groups was assessed before the surgery and at discharge (day 10 after the TKA). Measures included AROM, which was evaluated by a goniometer with the patient in the supine position. Knee flexion was measured with the hip at a 90° flexion. The axis of the device was placed in line with the center of the knee. The fixed arm aligned with the greater trochanter and the mobile arm with the lateral malleolus. Clinical evaluation was performed using KSS. Clinician-reported measures included pain, total range of flexion, flexion contracture, extension lag, stability, and alignment. The patient-reported score evaluated the patient’s mobility (walking distance, stair climbing, and using walking aids). The perceived, subjective functional status of patients was assessed using the WOMAC score. The scale assessed the patients’ perception of pain, stiffness, and ability to perform the activities of the daily life (ADL) and was performed as a self-reported questionnaire.

The Bioethics Committee of Poznan University of Medical Sciences approved the study. All procedures followed were in accordance with the ethical standards of the responsible committee on human experimentation and with the Helsinki Declaration of 1975, as revised in 2008. Informed consent was obtained from all patients for being included in the study.

### Statistical analysis

The results were analyzed with the Statistica Software version 13.1. Descriptive statistics were reported as means, standard deviations (SD), median, minimum, and maximum. The Shapiro–Wilk test was used to assess the normality of distributions in the test score. The independent *t*-test, Welch test (when the variance was different), and non-parametric Mann Whitney *U* test were conducted to compare the differences between the experimental and control group. The paired *t*-test and Wilcoxon’s signed ranks test were used to compare the pre- and post-intervention outcomes. The *p* values of less than 0.05 were considered statistically significant.

## Results

Preoperatively, there were no statistically significant differences between the groups in the demographic parameters or active knee flexion (Table 1). Differences were found, however, in the terms of the KSS scores determined at the baseline (Table 2). A comparison of the patients in the experimental and control group, conducted at discharge from hospital, revealed statistically significant differences in terms of the functional KSS and WOMAC scores (Table 3). There were no significant differences in terms of the range of active knee flexion or total KSS score. Moreover, in both groups, the active knee flexion at discharge was significantly smaller than the pre-operative flexion (Table 4). Additionally, patients in both groups showed worse WOMAC scores than before surgery. However, there was no significant improvement in the KSS score (Table 4).

**Table 1:** Baseline demographics of the patients

Variable	Experimental group (*n* = 43)	Control group (*n* = 37)	*p* Value
Sex (f/m)	30/13	32/5	-
Age (years)	70.5 ± 6.1 (58–85)	71.5 ± 6.1 (57–82)	0.503
Weight (kg)	79.2 ± 11.1 (56.4–105)	79.5 ± 10.4 (60–109.6)	0.896
Height (cm)	160.2 ± 9.0 (143–178)	156.9 ± 7.8 (140–175)	0.087
BMI (kg/m^2^)	30.8 ± 3.3 (26.1–39.5)	31.3 ± 3.6 (22.6–37.7)	0.525

Values are presented as a mean ± SD. Ranges of values are shown in brackets

Independent *t*-test

**Table 2:** Baseline clinical characteristics of the patients

Variable	Experimental group (*n* = 43)	Control group (*n* = 37)	*p* Value
Active knee flexion (°)	99.1 ± 20.9 (30–125)	97.7 ± 28.2 (0–140)	0.900*
KSS clinical (0–100)	66.1 ± 15.0 (35–94)	59.2 ± 21.4 (10–89)	0.247*
KSS functional (0–100)	75.8 ± 15.0 (40–100)	47.4 ± 23.1 (0–100)	0.000˟
KSS total	141.9 ± 25.0 (82–186)	106.6 ± 36.8 (10–180)	0.000*
WOMAC total (0–100)	57.5 ± 13.0 (25–85)	56.7 ± 4.6 (46–65)	0.722˟

Values ae presented as a mean ± SD. Ranges of values are shown in brackets

˟Welch test

*Mann Whitney *U* test

**Table 3:** Adjusted mean between-group differences at discharge

Variable	Experimental group (*n* = 43)	Control group (*n* = 37)	*p* Value
Active knee flexion (°)	82.3 ± 14.3 (40–100)	76.1 ± 22.2 (25–110)	0.325*
KSS clinical (0–100)	70.0 ± 15.0 (30–90)	73.5 ± 12.0 (35–92)	0.367*
KSS functional (0–100)	66.4 ± 8.1 (40–90)	62.2 ± 7.3 (50–80)	0.009*
KSS total	136.4 ± 19.3 (70–170)	135.7 ± 15.1 (95–167)	0.609*
WOMAC total (0–100)	38.6 ± 14.3 (3–67)	21.2 ± 15.7 (0–56)	0.000*

**Table 4:** Differences in measured variables change in both groups before and after surgery

Variable	Experimental group (*n* = 43)	Control group (*n* = 37)
Active knee flexion (°)	< 0.001	< 0.001
KSS clinical (0–100)	0.183	0.001
KSS functional (0–100)	0.001	< 0.001
KSS total	0.236	< 0.001ª
WOMAC total (0–100)	< 0.001ª	< 0.001ª

Paired *t*-test

ªWilcoxon’s signed ranks test

## Discussion

The concept of CPM, originally developed for promoting articular cartilage damage healing, evolved to remain a widely used adjunct to physiotherapy after TKA. Although numerous studies have been carried out on the effect of CPM after TKA, controversies still exist. In this study, we investigated whether CPM impacts early recovery outcomes in patients after TKA.

Both the experimental and control groups were homogenous in terms of age, weight, height, and BMI. In the initial assessment, statistically significant difference was noticed in the functional and total KSS scores in favor of the experimental group. At discharge, the experimental group showed higher scores in the functional KSS and WOMAC scales. However, comparing both groups before and after surgery, it could be seen that while in the control group, there was no improvement in all of the examined parameters (AROM, KSS clinical, KSS functional, KSS total, WOMAC total), in the experimental group, there was no statistically significant improvement only in the KSS clinical and KSS total scores. The reason for this could be the high score reached by this group in the initial assessment of the KSS total, and therefore a better effect could be achieved in patients with a lower initial score. Herbold et al. [12] compared two groups (CPM and no CPM) with initial knee flexion less than 75° and reported significant improvement in all examined parameters; however, the difference between the groups was not statistically significant. Brunn-Olsen et al. [13] reported a similar observation, with no difference between the CPM and no CPM groups either after one week or three months of treatment. Joshi et al. [14] pointed out that while there were no clinical benefits of using CPM, some additional costs were generated, and therefore this should no longer be considered as a standard procedure. A comparable observation was made by Boese et al. [10] who discontinued the routinely use of CPM after the TKA.

Nonetheless, other researchers found a positive effect of CPM on the TKA patients’ recovery. Bakirhan et al. [15] compared the course of treatment in two groups: high angle CPM (day 1: 60–70° + 10°/day) and low angle CPM (day 1: 30–40° + 10°/day). They observed that in the first group, results were better in terms of length of hospitalization and the Iowa Level of Assistance Scale (ILAS) scores. The only exception was gait speed, where a low angle CPM group achieved better results. These authors however suggested that this could be the effect of the longer hospitalization period of this group.

Based on the observation that one third of the patients were able to reach 120° of ROM in 3 days, the next third in four to seven days and the rest in more than seven days of hospitalization. Liao et al. [16] underlined the importance of applying ROM of CPM individually to all patients from the first day after the operation. Werner et al. [17] presented the results of CPM in a group of patients after Manipulation Under Anesthesia (MUA) performed due to the poor improvement of flexion (< 90° after 6 weeks post-op). After MUA, steroid drugs and aggressive CPM protocol (110° from day 1, 22 h/day during the first week, 8 h/day during the second week), a significant increase of ROM persisted after seven weeks (115°) and lasted for 74 weeks (116°).

The results of this study support these observations, although in most publications, no significant influence of CPM on the effects of rehabilitation after TKA was reported. Most of the studies, however (including our own), deny the validity of CPM usage, since they were constructed as a comparison of two groups—with or without CPM. Perhaps this might be a significant limitation of these studies, since Liao et al. [16] suggested that there is only a limited group of patients that react positively to CPM therapy. Other limitation of our study is the fact that our conclusions may be difficult to extrapolate, since there are a variety of implants used in primary TKA, different surgical techniques and analgesic standards of care. Therefore, our results are limited to comparable population, diagnosed with OA, who underwent primary TKA and were subjected to similar standards of post-operative care.

## Conclusions

Application of CPM in an early post-op treatment of patients after TKA did not have a significant impact on the clinical and functional evaluation after ten days of treatment. There were also no significant differences between the experimental and control groups in terms of range of motion, clinical knee score, and functional activity. Our results indicate that according to physiotherapeutic protocols, application of CPM did not restore the knee range of motion when compared to pre-op value, although at a discharge from the hospital, statistically significant differences in terms of functional KSS and WOMAC scores were recorded, and there was a significant beneficial effect of CPM on the subjective assessment of pain level, joint stiffness, and functional ability.

Because there is only a limited number of patients that react positively to the CPM therapy to achieve better therapeutic effects, further research should be conducted to identify these patients, in order to adjust physiotherapeutic procedure to individual patients.

## Data Availability

The datasets generated during and/or analyzed during the current study are available from the corresponding author on reasonable request.

## References

[1] Rankin EA, Alarcòn GS, Chang RW, Cooney LM, Costley LS, Delitto A, Deyo RA, Donaldson SK, Hochberg MC, MacLean CH, Yelin EH, Marciel K (2004). NIH Consensus statement on total knee replacement. J Bone Joint Surg Am.

[2] O’Driscoll SW, Giori NJ (2000). Continuous passive motion (CPM): theory and principles of clinical application. J Rehabil Res Dev.

[3] Salter RB, Simmonds DF, Malcolm BW, Rumble EJ, MacMichael D, Clements ND (1980). The biological effect of continuous passive motion on the healing of full-thickness defects in articular cartilage. An experimental investigation in the rabbit. J Bone Joint Surg Am.

[4] Beaupré LA, Davies DM, Jones CA, Cinats JG (2001). Exercise combined with continuous passive motion or slider board therapy compared with exercise only: a randomized controlled trial of patients following total knee arthroplasty. Phys Ther.

[5] Chen B, Zimmerman JR, Soulen L, DeLisa JA (2000). Continuous passive motion after total knee arthroplasty: a prospective study. Phys Ther.

[6] Lau SK, Chiu KY (2001). Use of continuous passive motion after total knee arthroplasty. J Arthroplasty.

[7] MacDonald SJ, Bourne RB, Rorabeck CH, McCalden RW, Kramer J, Vaz M (2000). Prospective randomized clinical trial of continuous passive motion after total knee arthroplasty. Clin Orthop Relat Res.

[8] Montgomery F, Eliasson M (1996). Continuous passive motion compared to active physical therapy after knee arthroplasty: similar hospitalization times in a randomized study of 68 patients. Acta Orthop Scand.

[9] Johnson DP (1990). The effect of continuous passive motion on wound healing and joint mobility after knee arthroplasty. J Bone Joint Surg Am.

[10] Boese CK, Weis M, Phillips T, Lawton-Peters S, Gallo T, Centeno L (2014). The efficacy of continuous passive motion after total knee arthroplasty: a comparison of three protocols. J Arthroplasty.

[11] Brosseau L, Milne S, Wells G, Tugwell P, Robinson V, Casimiro L, Pelland L, Noel MJ, Davis J, Drouin H (2004). Efficacy of continuous passive motion following total knee arthroplasty: a metanalysis. J Rheumatol.

[12] Herbold JA, Bonistall K, Blackburn M, Agolli J, Gaston S, Gross C, Kuta A, Babyar S (2014). Randomized controlled trial of the effectiveness of continuous passive motion after total knee replacement. Arch Phys Med Rehabil.

[13] Brunn-Olsen V, Heiberg K, Mengshoel AM (2009). Continuous passive motion as an adjunct to active exercises in early rehabilitation following total knee arthroplasty – a randomized controlled trial. Disabil Rehabil.

[14] Joshi RN, White PB, Murray – Weir M, Alexiades MM, Sculco TP, Ranawat AS,  (2015). Prospective randomized trial of the efficacy of continuous passive motion post total knee arthroplasty: experience of the Hospital for Special Surgery. J Arthroplasty.

[15] Bakirhan S, Ünver B, Karatosun V (2015). Effects of two different continuous passive motion protocols on the functional activities of total knee arthroplasty inpatients. Acta Orthop Traumatol Turc.

[16] Liao CD, Huang YC, Lin LF, Chiu YS, Tsai JC, Chen CL, Liou TH (2016). Continuous passive motion and its effects on knee flexion after total knee arthroplasty in patients with knee osteoarthritis. Knee Surg Sports Traumatol Arthrosc.

[17] Werner S, Jacofsky M, Kocisky S, Jacofsky D (2015). A standarized protocol for the treatment of early postoperative stiffness following total knee arthroplasty. J Knee Surg.

